# Southern African guidelines on the safe use of pre-exposure prophylaxis in persons at risk of acquiring HIV-1 infection

**DOI:** 10.4102/sajhivmed.v17i1.455

**Published:** 2016-03-15

**Authors:** Linda-Gail Bekker, Kevin Rebe, Francois Venter, Gary Maartens, Michelle Moorhouse, Francesca Conradie, Carole Wallis, Vivian Black, Beth Harley, Robyn Eakles

**Affiliations:** 1 The Desmond Tutu HIV Centre, University of Cape Town, South Africa; 2 Anova Health Institute, Johannesburg, South Africa; 3 Wits Reproductive Health and HIV Institute, Johannesburg, South Africa; 4 Department of Medicine, University of Cape Town, South Africa; 5 Right to Care and Clinical HIV Research Unit, University of the Witwatersrand, South Africa; 6 BARC, Johannesburg, South Africa; 7 Lancet Laboratories, Johannesburg, South Africa; 8 City Health, City of Cape Town, South Africa

## Abstract

The Southern African HIV Clinicians Society published its first set of oral pre-exposure prophylaxis (PrEP) guidelines in June 2012 for men who have sex with men (MSM) who are at risk of HIV infection. With the flurry of data that has been generated in PrEP clinical research since the first guideline, it became evident that there was a need to revise and expand the PrEP guidelines with new evidence of safety and efficacy of PrEP in several populations, including MSM, transgender persons, heterosexual men and women, HIV-serodiscordant couples and people who inject drugs. This need is particularly relevant following the World Health Organization (WHO) Consolidated Treatment Guidelines released in September 2015. These guidelines advise that PrEP is a highly effective, safe, biomedical option for HIV prevention that can be incorporated with other combination prevention strategies in Southern Africa, given the high prevalence of HIV in the region. PrEP should be tailored to populations at highest risk of HIV acquisition, whilst further data from studies in the region accrue to guide optimal deployment to realise the greatest impact regionally. PrEP may be used intermittently during periods of perceived HIV acquisition risk, rather than continually and lifelong, as is the case with antiretroviral treatment. Recognition and accurate measurement of potential risk in individuals and populations also warrants discussion, but are not extensively covered in these guidelines.

## Introduction

### Pre-exposure prophylaxis

Pre-exposure prophylaxis (PrEP) involves taking a pharmaceutical agent prior to an exposure to prevent an outcome (e.g. infection by a microbe, such as malaria). PrEP for HIV involves the use of antiretroviral (ARV) medications to prevent HIV infection. Research into the use of existing and novel PrEP agents, as well as various delivery systems, including topical gels and rings (microbicide) and oral (tablet) and long-acting injectable formulations, is ongoing.

Tenofovir (TDF) and tenofovir/emtricitabine (TDF/FTC) in a single tablet fixed-dose combination (FDC) are the oral ARV agents used in oral PrEP studies to date. The present guidelines support the use of TDF/FTC in combination for effective PrEP. TDF-containing PrEP is recommended by the World Health Organization (WHO) for people at substantial risk of HIV infection.^1^ In December 2015, the TDF/FTC combination pill was approved for use as PrEP by the Medicine Control Council, in combination with safer sexual practices.^2^

The aim of the this PrEP guideline is to:

- explain what PrEP is
- outline current indications for its use
- outline steps for appropriate user selection
- provide guidance to monitor and maintain PrEP users.

PrEP is indicated for HIV-negative men who have sex with men (MSM), transgender persons, heterosexual men and women (including adolescents) and people who inject drugs (PWID), who are assessed to be at high risk for HIV acquisition. PrEP should be used as part of a package of HIV prevention services (which may include regular HIV testing, condoms, lubrication, contraception, sexually transmitted infection [STI] management and risk reduction counselling). PrEP is also applicable to individuals at risk of HIV acquisition because they are unwilling or unable to consistently use male or female condoms, especially if in serodiscordant relationships. The user must be counselled on ongoing pregnancy and STI risk. PrEP can also be effective as part of a broader prevention package for people who use and inject drugs (PWID) in the comprehensive setting of needle and syringe exchange and opioid substitution programmes and access to ART for injecting networks. Harm reduction is an extensively proven HIV prevention intervention for PWID, but is not discussed further in these guidelines.

### Recommendations

Daily PrEP may be used intermittently during periods of perceived HIV acquisition risk, rather than continually and lifelong, as is the case with ARV treatment. HIV testing, estimation of creatinine clearance, pregnancy screening, and STI and hepatitis B screening are recommended as baseline investigations. Hepatitis B vaccination should be offered to susceptible individuals. Daily oral tenofovir/emtricitabine (TDF/FTC) as FDC, along with effective use support, can then be prescribed for eligible users. PrEP should not be given to those with abnormal renal function, nor should it be commenced in individuals with acute viral symptoms. An initial three-drug post-exposure prophylaxis (PEP) approach may be used whilst confirming HIV-negative status in an individual presenting with acute viral symptoms and a concomitant history of recent potential HIV exposure. An alternative HIV risk reduction method should be used until HIV-negative status is confirmed. Once HIV-negative status is confirmed, switching to PrEP can be discussed. Three-monthly follow-up visits to assess HIV status, pregnancy, tolerance, renal function, adherence and ongoing eligibility is recommended. Six-monthly STI screens and annual creatinine levels to estimate creatinine clearance are also recommended. Hepatitis B vaccination should be provided to susceptible clients. Headache and gastro-intestinal symptoms with weight loss are relatively common although usually mild and self-limiting, occurring for the first 4–8 weeks after initiating PrEP. These can be managed with counselling support and provision of symptomatic relief. Although uncommon, ARV resistance is most likely to occur amongst those who initiate PrEP with undiagnosed acute HIV infection. There is ongoing potential for resistance development among those with sub-optimal PrEP use who become HIV-infected while on PrEP. PrEP, if taken correctly and consistently, will offer protection from HIV infection but not from other STIs or pregnancy, and clinicians should continue to support PrEP users to be aware of STI symptoms and other components of combination prevention. Research is ongoing to assess optimum dosing regimens, potential long-term effects and alternative PrEP medications. Recommendations for the use of PrEP among other at-risk individuals, and the components of these recommendations, will be informed by future evidence.

### Background

#### Development of pre-exposure prophylaxis

Tenofovir disoproxil fumarate (TDF) alone or in combination with emtricitabine (FTC) was chosen for the evaluation of PrEP because of its high level of activity in inhibiting HIV replication; its acceptable safety profile; its high barrier to generating resistant virus; and its low levels of side-effects.^3^ The protective activity of TDF and FTC has been shown in animal models, with best efficacy when both agents were used together.^4,5^ In clinical trials, however, it has been shown that the difference in efficacy between TDF/FTC and TDF alone is insignificant. The use of TDF monotherapy for HIV prevention has not been investigated in some key populations such as MSM.

The Global iPrEx trial was the first randomised controlled trial to report decreased risk of HIV acquisition amongst at-risk MSM and transgender persons.^6^ These findings were further confirmed in the IPERGAY and PROUD studies.^7,8^ IPERGAY used an ’on-demand’ dosing strategy. However, as yet there are no data to support such a dosing strategy in other at-risk populations, and the writing group recommends daily PrEP in all at-risk groups until further data emerge supporting ‘on-demand’ dosing.

The Partners PrEP and TDF2 trials were both conducted in Africa and showed high levels of protection with daily oral tenofovir-based PrEP in heterosexual men and women including those in serodiscordant couples.^9,10^

The Bangkok Tenofovir Study was conducted using tenofovir only as PrEP amongst PWID.^11^ The risk overall in the study was reduced by 49%, and by up to 74% amongst those with detectable levels of tenofovir in their blood.

To date there have been 10 randomised controlled trials of TDF-based PrEP reporting HIV outcomes. The studies have involved more than 17 000 people and have demonstrated an overall reduction in HIV acquisition risk of 51% (women RR 0.57 [95% CI 0.34–0.94] and men RR 0.38 [95% CI 0.2–0.6]). Three studies in which there was high adherence to the study product (> 70% of drug detection) showed PrEP was most efficacious but HIV infection was also significantly reduced in those studies in which drug detection levels were moderate (41% – 70% detection). Unfortunately in the two studies with lowest adherence (< 40% drug detection), involving heterosexual women in southern and east Africa, PrEP had no effect.^12,13^ The reasons for the particularly low uptake and use of oral PrEP in these two studies have been speculated on elsewhere and a range of potential reasons have been suggested, including structural, behavioural and/or psychological factors. Unfortunately this has led to some controversy around the effectiveness of oral PrEP in black African women. It is important to note, however, that two of the three studies considered by the US Food and Drug Administration (FDA) prior to licensure of PrEP as a prevention modality included women from Uganda, Kenya and Botswana. What does emerge clearly from all these studies is the fact that protection strongly correlates with adherence to the study drug, assessed in most studies by random tenofovir drug levels.

**FIGURE 1:**
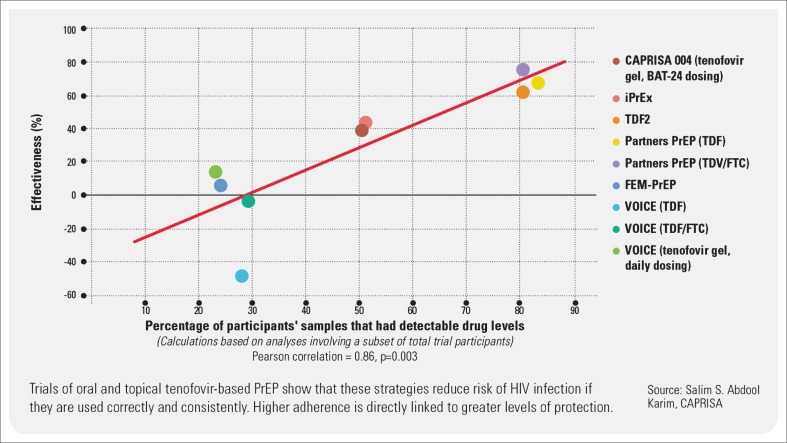
Effectiveness and adherence in trials of oral and topical tenofovir-based prevention.

Additional open-label demonstration projects and implementation science studies amongst different at-risk populations are ongoing (http://www.avac.org/ht/a/GetDocumentAction/i/3113) and confirm high rates of protection amongst the individuals with best effective use. No RCTs of tenofovir-based PrEP are currently underway, although alternative ARVs, new longer-acting formulations and alternative topical applications are planned or are still in earlier phase studies (Figure 1).

## MSM, transwomen and HIV in Southern Africa

MSM is a behavioural term that describes MSM, regardless of social identity (gay, bisexual, heterosexual) or whether they also have sex with women.^14^ MSM and transgender persons have been shown to be at disproportionately high risk of HIV acquisition and transmission.^15,16^ Biological susceptibility (efficiency of rectal HIV transmission), behaviours (including condomless sex, anal intercourse and multiple partners) as well as structural and social factors (including homophobia and discrimination) have been associated with increased vulnerability to HIV.^16^ Condomless receptive anal intercourse is the main risk factor for sexual transmission of HIV among MSM.^17^ The high concentration of rectal cells vulnerable to HIV-1 infection (macrophages, T-cells and dendritic cells) and the single-cell layer of rectal mucosa, results in a per-act risk for HIV transmission that is 10–20 times greater than unprotected vaginal intercourse.^17,18,19^

There is emerging and consistent evidence about the high HIV burden amongst MSM in Southern Africa.^20^ Unfortunately, little data exist on the trans populations in South Africa. HIV prevalence amongst MSM sampled in cross-sectional surveys in South Africa has ranged from 10% – 50%.^21,22,23,24^ However, owing to the lack of accurate population size estimates, it is hard to assess attributable risk.^25^ A 2009 modelling study on the modes of HIV transmission in South Africa estimated that 8% of all new HIV infections in South Africa occur among MSM.^26^ High-risk sexual practices (including unprotected anal intercourse, multiple and concurrent partnerships, and sex work) and limited knowledge about HIV and substance use (alcohol, methamphetamines and heroin) have been associated with increased risk for HIV infection amongst MSM in South Africa.^15,22,23,24,27,28,29^

Many MSM also have female sexual partners. Almost half (49%) of the participants in a Soweto-based MSM study reported recent female sexual partners.^23^ Homophobia, stigma and discrimination (including criminalisation of same-sex behaviours in some Southern African countries), healthcare worker ignorance (about MSM and transgender vulnerability to HIV and appropriate management of MSM clients) and the heterosexual focus of the HIV response have been contributing factors to the failure of Southern African public health services to address the health needs of MSM and transgender persons.^15,25,30,31,32,33,34,35,36,37^

### Motivation for a pre-exposure prophylaxis guideline

The initial iPrEx trial results contributed to an earlier version of these guidelines and the development of interim guidance on the use of PrEP amongst MSM by the United States Centers for Disease Control and Prevention.^38^ These revised guidelines include all at-risk populations consistent with all accrued clinical evidence, the CDC guidelines^39^ and recent (2015) WHO recommendations.^1^ Southern African guidelines will assist practitioners who may be considering, or are already, prescribing PrEP to people at risk.

### Identification of potential pre-exposure prophylaxis users

Providers should educate and counsel potential PrEP users about PrEP and conduct an individualised risk-benefit assessment to assess eligibility (Box 1). The eligibility assessment requires that providers have developed sufficient client rapport to effectively assess risk based on these self-reported behaviours.

BOX 1 Risk behaviour assessment for sexual HIV acquisition.

**Risk behaviour assessment for MSM and transwomen**

In the past 6 months:

1. Have you had sex with men, women or both?
2. How many men have you had sex with?
3. How many times did you have receptive anal sex with a man who was not wearing a condom?
4. How many of your partners were HIV-positive or of unknown HIV status?
5. With these positive/unknown status partners, how many times did you have insertive anal sex without wearing a condom?

**Risk behaviour assessment for heterosexual men and women**

In the past 6 months:

1. Have you had sex with men, women or both?
2. How many men/women have you had sex with?
3. How many times did you have vaginal or anal sex when neither you nor your partner wore a condom?
4. How many of your partners were HIV-positive or of unknown HIV status?
5. With these positive/unknown status partners, how many times did you have vaginal or anal sex without wearing a condom?

*Source*: Adapted from HHS guideline (Preexposure prophylaxis for the prevention of HIV infection in the United States – 2014 clinical practice guideline)

### Indications for the use of pre-exposure prophylaxis

PrEP should be considered for people who are HIV-negative and at significant risk of acquiring HIV infection (see Boxes 2 and 3). PrEP may be suitable for:

- any sexually active HIV-negative *MSM or transgender person* who wants PrEP
  ▪ those with HIV-positive sexual partner(s) who are not confirmed virologically suppressed
  ▪ partner(s) of unknown HIV status
  ▪ recent STI
  ▪ multiple sexual partners
  ▪ history of inconsistent or no condom use
  ▪ commercial sex work
  ▪ recurrent PEP users
  ▪ history of sex whilst under the influence of alcohol or recreational drugs
- *heterosexual* women and men who want PrEP, targeting especially
  ▪ those with HIV-positive sexual partner(s) who are not confirmed virologically suppressed
  ▪ partner(s) of unknown HIV status
  ▪ recent STI
  ▪ multiple sexual partners
  ▪ history of inconsistent or no condom use
  ▪ commercial sex work
  ▪ serodiscordant couples trying to conceive
  ▪ recurrent PEP users
  ▪ history of sex whilst under the influence of alcohol or recreational drugs
- people who inject *drugs*
  ▪ HIV-negative PWID with HIV-positive/unknown status injecting partner(s)
  ▪ share injecting needles and drug preparation equipment
- all of the above groups include *adolescents* and *sex workers*, which each constitute special groups meriting specific consideration
  ▪ especially vulnerable are young MSM and adolescent girls.

BOX 2 Indications for the use of pre-exposure prophylaxis.

1. Any sexually active HIV-negative **MSM or transgender person** especially:
  - those with HIV-positive sexual partner(s) who are not confirmed virologically suppressed
  - partner(s) of unknown HIV status
  - recent STI
  - multiple sexual partners
  - history of inconsistent or no condom use
  - commercial sex work
  - recurrent PEP users
  - history of sex whilst under the influence of alcohol or recreational drugs.
2. **Heterosexual** women and men especially:
  - those with HIV-positive sexual partner(s) who are not confirmed virologically suppressed
  - partner(s) of unknown HIV status
  - recent STI
  - multiple sexual partners
  - history of inconsistent or no condom use
  - commercial sex work
  - serodiscordant couples trying to conceive
  - recurrent PEP users
  - history of sex whilst under the influence of alcohol or recreational drugs.
3. People who inject **drugs:**
  - HIV-negative PWID with HIV-positive/unknown status injecting partner(s)
  - share injecting needles and drug preparation equipment.
4. All of the above groups include **adolescents** and **sex workers**, which each constitute special groups meriting specific consideration:
  - Especially vulnerable are young MSM and adolescent girls.

BOX 3 Eligibility criteria for pre-exposure prophylaxis use.

**Eligibility criteria for PrEP use include:**

- anyone identified by the provider and client as being at high risk for HIV exposure (see text box on indications for the use of PrEP)
- no contraindications to FTC/TDF FDC
- HIV-negative by routine rapid antibody test and not thought to be in the window period for HIV seroconversion
- absence of symptoms of acute HIV infection (recent acute viral illness) and, if symptoms reported, HIV-negative by 4th-generation HIV test or other HIV antigen test if available (this reduces, but doesn’t eliminate, the window period)
- willing and able to attend 3-monthly PrEP maintenance visits, inclusive of HIV counselling and testing, clinical review and safety monitoring procedures
- client understanding that the protection provided by PrEP is not complete, and does not prevent other STIs or unwanted pregnancies, and therefore PrEP should be used as part of a package of HIV prevention services (inclusive of condoms, lubrication, contraception, risk reduction counselling and STI management)
- recurrent use of PEP.

PrEP should be provided as part of a combination prevention package.

### Contraindications to pre-exposure prophylaxis

- HIV-1 infected or evidence of possible acute infection
- suspicion that patient might be in the window period for HIV testing following potential exposure
- adolescents < 35 kg or < 15 years of age who are not Tanner stage 3 or greater should not be given TDF
- poor renal function (estimated creatinine clearance < 60 mL/min)
- TDF should not be co-administered with other nephrotoxic drugs, for example, aminoglycosides
- unwilling or unable to return for 3-monthly HIV testing, counselling and safety monitoring visits
- pregnant or breastfeeding women^2^ (see section below entitled Special clinical considerations).

## Initiation of pre-exposure prophylaxis

Steps for the screening/baseline visit, PrEP initiation visit and maintenance of PrEP are described below.

### Baseline investigations

After documenting eligibility and motivation for PrEP use, mandatory baseline investigations should be completed (Table 1). The minimum package of tests offered should include:

**TABLE 1:** Mandatory baseline investigations for pre-exposure prophylaxis initiation.

Screening	Method
HIV infection	Laboratory ELISA preferably - fourth generation rapid if ELISA not available
Renal function	eGFR > 60 mL/min
Hepatitis B screen	Surface antigen (HBsAg) Antibody to surface antigen (HBsAb)
STI screen	Symptomatic screen Examination if indicated Urine dipstix for urethritis Serological screening for syphilis (rapid or laboratory) Full STI panel if resources allow
Pregnancy screen	Rapid pregnancy test or beta HCG

- assessment of HIV status: Preferably use laboratory ELISA. If not available, use a fourth generation rapid test. Always repeat a positive test with a confirmatory test. If a fourth generation test is not available, defer PrEP commencement, use rapid test available in facility and repeat in 2–4 weeks whilst counselling client on risk reduction. If this subsequent test is negative and there is no reported recent risk and no symptoms, PrEP may be initiated
- where history of recent exposure, consider PEP per guidelines before initiating PrEP
- check creatinine and calculate creatinine clearance
- syndromic STI screen or, if resources allow, a full STI panel regardless of symptoms. Treat any STIs detected as appropriate, according to the relevant local guidelines
- hepatitis B surface antigen and antibody – if both negative, vaccinate against hepatitis B virus (HBV). Acute or chronic HBV is not a contraindication to PrEP but monitoring of LFTs is advised, especially if considering cycling off PrEP
- pregnancy screen.

Condoms and condom-compatible lubrication should be provided, and arrangements made for follow-up.

#### Problems with using rapid HIV tests in the field

There are several point of care (POC) or rapid tests available for detection of HIV in the field. Rapid and accurate diagnosis of HIV is needed in the setting of PrEP. This on-site detection of HIV removes the risk of loss to follow-up and decreases the time of individuals taking PrEP if they are HIV-positive. A major downfall of POC HIV diagnostic tests is that they have a larger window period for HIV diagnosis at PrEP commencement than molecular-based assays and are also more prone to inaccurate reporting. There is a growing need to ensure that optimal conditions for rapid HIV testing are followed, ensuring that the highest level of quality and sensitivity is achieved.^40^ This includes improved training of both technical and clinical staff; improvement of testing space in the clinic; improvement of laboratory information systems for management of the patient results; and the need to implement external quality programmes covering all steps of the assay.

### Implementing pre-exposure prophylaxis

The PrEP initiation visit should take place no longer than one month after the screening/baseline visit. At this visit, review lab ELISA results, repeat the rapid HIV test and do a review for acute viral symptoms. Review results from baseline investigations and confirm that estimated creatinine clearance is > 60 mL/min. Commence HBV vaccination if susceptible and provide STI treatment as required (refer to latest national guidelines). Educate the user about potential PrEP side-effects and their management, as well as signs and symptoms of acute HIV infection (and the need to return for urgent HIV testing). HIV testing should be repeated every 3 months.

Initiate an effective PrEP use plan (Box 4) and provide a one-month TDF/FTC 300/200 mg (FDC) prescription (one tablet orally daily) together with a one-month follow-up date (Table 2). Those individuals with a recent high-risk exposure (e.g. a sex worker) can transition from PEP to PrEP.

BOX 4 What if users ask about stopping condom use while on pre-exposure prophylaxis?

1. Do not be judgemental about patient preferences.
2. Explain that this is a valid choice but there are potentially negative consequences.
3. Stress that PrEP prevents HIV but not STIs.Stress that PrEP prevents HIV but not pregnancy.
4. Confirm a regular STI screening and management plan.
5. Confirm an effective and acceptable contraception plan where indicated.
6. Vaccinate against all vaccine-preventable STIs, e.g. hepatitis A and B and HPV where possible.

**TABLE 2:** Summary of pre-exposure prophylaxis visits and procedures.

Visit	Recommended procedures
Screening	Educate about the risks and benefits of PrEP Assess risk and eligibility Conduct HIV counselling and testing, serum creatinine level, hepatitis B and STI screen, pregnancy test Contraceptive counselling and offer services Arrange follow-up visit
PrEP initiation	Conduct HIV counselling and testing Confirm eligibility (including investigation results and creatinine clearance calculation) Commence hepatitis B vaccination if indicated Provide STI treatment if indicated Pregnancy test Educate client about PrEP side-effects and management Educate client about signs and symptoms of acute HIV infection Discuss behaviours that promote bone health, such as weight-bearing exercise and avoiding alcohol, tobacco and recreational drugs Initiate a medication effective use plan Provide condoms and lubricant Contraceptive counselling and offer services as appropriate Provide one-month TDF/FTC (FDC) prescription and follow-up date
One-month follow-up	PrEP initiation visit, PLUS: Assess tolerability, side-effects and effective use Actively manage side-effects Measure serum creatinine and calculate creatinine clearance Contraceptive services Provide three-month TDF/FTC (FDC) prescription and follow-up date
Four-month follow-up and 3-monthly maintenance visits	Repeat procedures done at one-month follow-up Measure serum creatinine and calculate creatinine clearance at four-month follow-up, and 12-monthly thereafter Conduct 6-monthly STI screen, including urine dipstix and rapid syphilis test Complete hepatitis B immunisation at 6 months

FDC, fixed-dose combination; FTC, emtricitabine; PrEP, pre-exposure prophylaxis; STI, sexually transmitted infection; TDF, Tenofovir.

It is important to bear in mind that MSM initiating PrEP need 7 days of daily dosing to reach adequate anal/rectal tissue levels, whilst women need up to 20 days of daily dosing to achieve protective vaginal tissue levels of PrEP drugs. During this period, other protective precautions must be used, such as abstinence or condoms. This should also be borne in mind in users who stop and start PrEP according to their periods of risk. PrEP medications should be continued for 28 days after the last potential HIV exposure in those wanting to cycle off PrEP.

## Risk reduction counselling

Risk-reduction counselling is a behavioural intervention that attempts to decrease an individual’s chances of acquiring HIV and other STIs,^41^ and should be implemented together with effective use counselling and contraceptive counselling at follow-up visits for PrEP users. The main objective of risk-reduction counselling is for clients to set a realistic goal for behaviour change that could reduce their risk of contracting HIV. This is most effective when it is non-prejudicial and user-centred. Risk reduction counselling can be provided by any trained healthcare provider and should address the following points:

- explore the context of the user’s specific sexual practices, and assist in recognising which of their behaviours are associated with higher risk of HIV infection. Clinicians should also be aware that clients might not always perceive their own risk, or may be in denial about it
- identify the sexual health protection needs of the user and reflect on what their main concerns appear to be
- strategise with the user on how they can manage these concerns or needs
- agree on which strategies the user is willing to explore, and guide the user to decide on how to implement the strategy (Box 4).

For effective use support, see Box 5.

BOX 5 ‘Adherence’ versus ‘effective use’.

These guidelines use the term ’effective use’ rather than ’adherence’. Adherence is often understood by healthcare workers, especially when applied to ARV treatment adherence, as life-long and at correct dosing intervals to ensure viral suppression. Oral PrEP must be taken, ideally daily, during times of HIV exposure risk, although there are some data suggesting that less than perfect adherence is still highly effective in MSM. There may be times when it would be appropriate to cycle off oral PrEP, for example when MSM move out of ’seasons of risk’, or when female sex workers (FSW) return home to visit family, taking a break from sexual activity. If appropriate consistent use of oral PrEP is measured with the same standard as we measure ARV treatment adherence, it may show up as lacking, when in fact the population at risk has used the drug effectively. The term ‘effective use’ is preferred to when discussing whether ARV-based prevention has been used successfully; this is akin to ’effective use of condoms’ as we seldom talk about condom adherence.

Adherence to daily PrEP medication, as shown in the iPrEx study and other PrEP trials, is a challenge. Effective use counselling should be implemented at each visit where PrEP prescriptions or distributions are made. In iPrEx, participants who took PrEP more consistently and had evidence of drug detection in their blood, had higher levels of protection than those who did not.^3^ These findings have been duplicated in other PrEP studies in different study populations. Users will need to be made aware of the fact that drugs only work if present at adequate levels in tissues and, preferably, that drug levels should be adequate before and after exposure to HIV has occurred. The use of cellphone reminders, pillboxes, and linking pill taking with a daily routine activity are currently being evaluated for their impact on improving PrEP effective use. Clinicians and clients could use any of these or other strategies to assist in maximising effective use (see Box 6 on tips to support effective use). Any trained healthcare worker can implement effective use counselling. A client-centred approach is recommended. Drug level testing for tenofovir levels in plasma is available, but is expensive. Drug level testing may be useful to assess effective use in the future.

BOX 6 Tips to support effective use.

Include user-focused effective use counselling at each contact. Provide a clear explanation of the benefits of effective use. In a neutral manner, ask if the user has any challenges that may make taking PrEP difficult. Also explore possible facilitators to pill taking. Include identified facilitators when developing strategies to improve effective use of PrEP.^42^

**Options to improve daily pill taking:**

- Use reminders (cellphone, alarm clock, diary, partner reminder).
- Link with daily activity (breakfast, brushing teeth).
- Use a pillbox.
- Food is **NOT** required for pill taking.
- Join an on-line support group, e.g. Facebook: PrEP Rethinking HIV Prevention or #wethebrave.

### Managing abnormal screening results

Clients with abnormal renal function (estimated creatinine clearance < 60 mL/min) should not be placed on PrEP. An abnormal estimated creatinine clearance result could be rechecked after 2 weeks and, if renal function returns to normal and other PrEP eligibility criteria are met, PrEP may be initiated. Those who are susceptible to hepatitis B should be immunised. Clients with acute or chronic hepatitis B can be safely initiated onto PrEP but may require LFT monitoring.^6^ Clients with a history of pathological bone fracture, a family history of osteoporosis, or decreased bone mineral density on DEXA scanning, should be educated on ways to improve bone health, such as weight-bearing exercise and avoiding alcohol, tobacco and recreational drugs.^43^ Clients who are ineligible for PrEP require support to access other prevention options (see HIV Prevention text box). Treat STIs syndromically as per national guidelines.^44^ Consider empiric gonorrhoea and chlamydia treatment for MSM who are highly sexually active, even in the absence of symptoms (especially where STI laboratory screening is not feasible). Most MSM with gonorrhoea and chlamydial infection are more likely to be asymptomatic than symptomatic.^45,46^ and can be managed in line with STI treatment guidelines (refer to latest national guidelines).

### Safety monitoring and maintenance

PrEP users require an initial one-month follow-up to assess ongoing eligibility, tolerance, safety and effective use. Hepatitis B vaccination and STI treatment (as appropriate), condoms and condom-compatible lubricant, contraceptive services, risk reduction counselling, effective use support, a 3-month prescription for TDF/FTC FDC and a follow-up date should be provided at this visit. Thereafter, 3-monthly visits are recommended (Table 3). Check the creatinine at the first-month and fourth-month visits, and thereafter 12-monthly. Check rapid HIV every 3 months.

**TABLE 3:** Hepatitis B immune status and pre-exposure prophylaxis eligibility.

Hepatitis B surface antigen (HBsAg)	Hepatitis B surface antibody (HBsAb)	Action
Negative (-)	Negative (-)	Start PrEP, vaccinate concurrently
Negative (-)	Positive (+)	Start PrEP, no vaccine needed
Positive (+)	N/A	Refer for evaluation

N/A, not applicable; PrEP, pre-exposure prophylaxis.

Each visit should be viewed as an opportunity for counselling and risk assessment. Discuss with the user at each visit whether PrEP is still needed. Emphasise the importance of effective use of PrEP to gain maximum ongoing benefit from PrEP.

By mutual agreement, PrEP should be stopped if: HIV test is positive; the client no longer meets eligibility criteria; the client does not need PrEP; the client feels that adherence to PrEP is too onerous; or it is perceived by the clinician that the risks of PrEP outweigh potential benefits. Users who are ineligible for PrEP require support to access other prevention options (see HIV prevention text box).

PrEP users should be reminded that PrEP is not conceptualised as a life-long therapy. It should be used while there is risk of sexual or other exposure to HIV. PrEP users are therefore expected to cycle on and off PrEP as dictated by their current level of sexual risk. A reminder should be given that, if restarting PrEP, adequate protection only occurs after 7 days of dosing for anal sex and 20 days of dosing for vaginal sex or needle risk in PWID. HIV-negative status should be confirmed before restarting PrEP. If stopping PrEP, medication should be taken for 28 days after the last potential exposure to HIV.

### Managing abnormal follow up visit results

PrEP should be stopped if estimated creatinine clearance < 60 mL/min. Repeat creatinine clearance should be rechecked after 2 weeks; if renal function returns to normal and other PrEP criteria are met, PrEP may be restarted. STIs should be treated syndromically (refer to latest national guidelines).

## Risks and side-effects

### Antiretroviral resistance

At the time of writing the guideline, the only HIV resistance documented to date amongst PrEP users has been amongst clients who initiated PrEP when they were already HIV infected (during acute HIV infection). Predictably, the FTC resistance mutation M184V was the first to occur.^3^ To prevent the risks of developing ARV resistance, clinicians must focus on not commencing or reinitiating PrEP after a break, during acute HIV infection (Box 7).

BOX 7 Strategies to reduce the likelihood of antiretroviral resistance.

**Feasibly exclude acute HIV infection before initiating PrEP by:**

- conducting antibody HIV testing before commencing or re-prescribing PrEP
- enquiring about pill taking patterns and whether any pills were missed
- among persons with a negative HIV antibody test, conducting a clinical screen to detect signs and symptoms of acute HIV infection – history of fever, sore throat, rash, joint pain, cough in the past month and a targeted examination (temperature, ENT and skin exam) (see Acute HIV infection text box)
- considering time period between last potential HIV exposure and window period of tests being used
- If symptoms or signs of acute HIV infection found:
  ▪ At screening: postpone PrEP until symptoms subside and rapid antibody test remains negative at 2–4 weeks’ follow-up
  ▪ At screening: do not initiate PrEP until follow-up HIV antigen/antibody testing (2–4 weeks) complete
  ▪ At follow-up: may elect to continue PrEP while awaiting results of follow-up HIV antigen/antibody testing (2–4 weeks) or may decide to withhold PrEP until follow-up tests available
  ▪ Note that, if PrEP has been taken consistently, breakthrough infection is unlikely. Withholding PrEP may put an effective user at greater risk for HIV acquisition
- Support client to maximise effective use and include effective use counselling at each visit
- Stop PrEP should requirements for PrEP eligibility not be fulfilled or if client recognises risk profile has altered or wishes to use a different combination of prevention
- Counsel client that recommencement will require all of the above steps again.

HIV testing should be done 3-monthly, and should be accompanied by an HIV exposure assessment, symptom screen and a targeted examination to exclude acute HIV infection (Box 8). HIV testing should also be repeated whenever symptoms of a viral illness are present. Clinicians should advise clients on the need for an HIV test before resuming PrEP if it was stopped, particularly if they have potentially been exposed to HIV during this period.

BOX 8 Acute HIV-infection.

Severity of the syndrome ranges from mild non-specific ‘viral’ or ‘flu-like’ symptoms to a severe infectious mononucleosis-like illness with immune dysregulation and transient profound CD4 depletion.^47,48^

**Symptom:**

- malaise
- anorexia
- myalgias
- headache
- sore throat
- sore glands
- rash.

**Sign:**

- fever, sweating
- generalised lymphadenopathy
- hepatosplenomegaly
- non-exudative pharyngitis
- orogenital herpetiform ulceration
- truncal rash (maculopapular or urticarial)
- viral meningitis
- Guillian-Barre syndrome
- *Pneumocystis* pneumonia†
- cryptococcal meningitis†
- oral/oesophageal candidiasis.

†, Extremely rare.

### Side-effects

There is a large TDF/FTC FDC safety database derived from millions of HIV-positive individuals receiving ART.^6^ In addition, the 17 000 individuals exposed during the clinical trials and an increasing number of individuals in demonstration projects confirm the extremely good safety profile of TDF/FTC FDC use in HIV-negative individuals.^6^

The major toxicities associated with TDF/FTC have been extremely rare in PrEP exposure to date. Minor side-effects have been relatively common, but mild and self-limiting (approximately 1 in 10 individuals in the first 1–2 months).

### Gastrointestinal side-effects

The side-effects related to TDF/FTC FDC use in PrEP trials (nausea, weight loss) were mostly self-limiting mild start-up symptoms (first month), but these may adversely affect persistent PrEP effective use. Supportive counselling and symptomatic treatment (anti-emetics) of these symptoms is often sufficient to assist the user to persist beyond the first month, after which the symptoms tend to subside. This may also be accompanied by mild headache and some malaise. Rates of other GIT symptoms (bloating, abdominal tenderness, flatulence) amongst PrEP trial participants who took TDF/FTC FDC were not significantly different from those who took placebo.^6^

### Renal toxicity

Modest, transient increases in serum creatinine have been noted in completed PrEP studies, but these did not persist after stopping PrEP nor recur on rechallenge. Proteinuria, decreasing glomerular filtration rate (GFR) and Fanconi’s syndrome have been described in the setting of ART, and decreased GFR has been described in the setting of PrEP but has not caused clinical harm.^6^ Renal function needs to be measured prior to commencement and monitored in clients using PrEP by measuring serum creatinine and calculating the estimated creatinine clearance. These parameters should be measured at baseline, at month 1, month 4 and then annually thereafter. Hypertensives, diabetics and those with existing glomerulonephropathies (if the benefit of PrEP is still deemed to outweigh clinical risk) should be monitored more frequently. TDF/FTC FDC-based PrEP should be avoided in patients who require the use of other nephrotoxic drugs, such as aminoglycosides for the treatment of drug-resistant tuberculosis (TB). Clients with creatinine clearance < 60 mL/min should not be placed on PrEP and, if found during maintenance, PrEP should be discontinued.

### Decreased bone mineral density

Decreases in bone mineral density associated with TDF and FTC/TDF FDC have been observed in completed PrEP trials. Decreases were less than those observed in HIV-infected individuals treated with the same drugs, and appeared to stabilise over time.^49,50^ No difference in fracture rates was seen. Recreational drugs (amphetamines and inhalant use) were associated with reductions in bone mineral density in HIV-negative MSM taking TDF, suggesting some synergistic impact.

### Hepatitis B management

TDF and FTC both have hepatitis B antiviral activity. The potential risk exists that exposure to these antivirals may treat unidentified chronic hepatitis B infection, with a consequent viral flare (rebound) upon drug withdrawal that can result in severe liver injury.^51^ This phenomenon has not been described with PrEP use to date. However, it is recommended that screening for hepatitis B surface antigen and antibodies occurs prior to PrEP commencement.

It is recommended that, if hepatitis B surface antigen (HBsAg) is positive, the client be investigated prior to commencement of short-term PrEP (Table 3). PrEP is not contra-indicated in those with HBV but we recommend that additional liver function monitoring should be performed. PrEP users with persistently elevated or abnormal liver function tests should be referred for assessment. A possible approach to those with chronic hepatitis B infection may be to prescribe long-term TDF/FTC FDC. Liver function tests should be checked after stopping PrEP in those with chronic hepatitis B infection. Users who are negative for both HBsAg and hepatitis B surface antibody (HBsAb) should commence a hepatitis B vaccine schedule. People with chronic hepatitis B infection may choose to continue using TDF and FTC to control their hepatitis, even if they do not require these drugs any longer for the indication of PrEP. Users with a history of injecting drug use should be screened for hepatitis C and, if positive, referred for further care.

### Other side-effects

Hyperpigmentation may occur as a side-effect to FTC. The clinician should explain that this is not harmful. Lamivudine (3TC) can be substituted but this will increase the pill burden, which may have an impact on effective PrEP use. PrEP studies to date have used either TDF or TDF in combination with FTC, rather than 3TC.

### Risk compensation

This term refers to the theoretical risk that individuals commencing PrEP will neglect other safer-sex measures, and put themselves at increased risk of HIV exposure. To date, evidence of this has not been borne out in PrEP trials. It may be, however, that during counselling it is apparent that a client may not be able to or simply cannot use condoms or other safer-sex modalities. In these cases, PrEP if used consistently during HIV exposure may significantly reduce HIV infection. Providers should gauge this during risk reduction and effective use counselling opportunities.

## HIV prevention package for pre-exposure prophylaxis users

The prevention of HIV acquisition requires a comprehensive approach, inclusive of a combination of biomedical and behavioural/psychosocial interventions tailored to individual needs. Where feasible, condoms and condom-compatible lubrication are key components of all HIV prevention packages, supported by contraceptive services, STI detection and treatment, appropriate use of ART (PEP), and counselling around the identification of high-risk practices and ways to circumvent or reduce risk. Individuals should be encouraged to understand what each component of the prevention package offers and, together with the provider, should devise the optimal package for their own lifestyle.

## Stopping pre-exposure prophylaxis

PrEP should be stopped: (1) whenever an HIV test is positive, (2) at client request, (3) for safety concerns (particularly if creatinine clearance < 60 mL/min) and (4) if the risks of PrEP outweigh the potential benefits. Ongoing linkage to appropriate HIV prevention services and contraceptive services should be encouraged, as well as the use of other HIV prevention strategies, as needed.

The duration of PrEP use may vary and individuals are likely to start and stop PrEP depending on their risk assessment at different periods in their lives – including changes in relationship status, behaviours and ability to adhere to a PrEP maintenance programme. Clients should be advised that an HIV test at minimum should be done before PrEP is recommenced. Clinicians may want to discuss the options of when to discontinue PrEP with their clients.

## Other notes for pre-exposure prophylaxis prescribers

### Pre-exposure prophylaxis will not suit all users

PrEP should be considered for clients who are most likely to benefit from this specific prevention strategy, ideally as part of a package of HIV prevention services (Box 9).

BOX 9 HIV prevention for pre-exposure prophylaxis users.

**General factors to consider:**

- accessibility of condoms and compatible water-based lubricant should be addressed
- no single HIV risk reduction intervention is likely to suit all users
- combinations of prevention options, tailored to address specific risks, should be offered (‘menu of prevention choices’), inclusive of biomedical and psychosocial/behaviour change interventions
- prevention options are likely to increase as new evidence becomes available.

**Biomedical:**

- male or female condoms and compatible lubrication
- access to frequent HIV testing
- early access to ART
- post-exposure prophylaxis
- pre-exposure prophylaxis
- voluntary medical male circumcision
- STI screening and treatment
- needle syringe exchange and opioid substitution therapy for people who inject drugs.

**Psychosocial:**

- education: risk and safer sex practices
- regular HIV counselling and screening
- reducing number of sex partners
- reducing alcohol and substance abuse
- addressing mental health needs
- couple counselling and programming
- harm reduction counselling and support for clients who use drugs.

### Pre-exposure prophylaxis usage requires commitment

Usage will require commitment from both the provider and the user to ensure success. Providers may need to be innovative in providing support to PrEP users and also find ways to make participation in a PrEP programme as easy and convenient as possible. This requires ensuring that structural, logistical barriers are minimised as much as possible and that participants are provided with an encouraging and positive approach from providers.

## Special clinical considerations

### Women who become pregnant or breastfeed on pre-exposure prophylaxis

HIV-negative women in serodiscordant relationships are at risk of acquiring HIV infection whilst trying to conceive through unprotected sex. Pregnancy itself is also associated with an increased risk of becoming infected with HIV. The use of PrEP around the time of conception and during pregnancy offers a means of protection to the uninfected partner. Unfortunately, data relating to the safety of PrEP specifically with regard to the developing foetus are limited, and consequently the onus is on the clinician to discuss potential risks and benefits of PrEP initiation or maintenance during pregnancy with the client.

PrEP trials involving heterosexual women excluded pregnant women from enrollment, and those who fell pregnant during the conduct of the study were discontinued from PrEP. One study of 46 uninfected women in serodiscordant relationships demonstrated no adverse effects on the pregnancy or cases of HIV transmission when TDF was used around the time of conception. There are several ongoing demonstration projects that will allow women to continue PrEP if they fall pregnant, which will provide some data to inform future recommendations. In addition, the Antiretroviral Pregnancy Registry shows no evidence of adverse outcomes amongst infants exposed to these medications when used as antiretroviral therapy *in utero*.

In serodiscordant couples, the infected partner should be initiated on ART and virologically suppressed, ideally for 6 months, before any attempts to conceive.

In South Africa, the use of TDF/FTC as PrEP in pregnant or breastfeeding women is contra-indicated. However, as the risk of seroconversion during pregnancy is high, the risks and benefits of PrEP should be discussed with potential PrEP users, allowing these women at high risk of HIV acquisition to make an informed decision regarding PrEP use.^2^

Exposure to PrEP via breast milk has not been extensively studied. However, HIV-negative babies born to HIV-positive mothers on PMTCT B+ programmes and lifelong ART are exposed to TDF/FTC. The risk of HIV infection against the risk of ARV exposure to the infant should frame a discussion with a potential PrEP user who is pregnant or is planning conception.

## The future of pre-exposure prophylaxis

Whilst recommendations for safe and effective PrEP use in correctly identified users to prevent HIV acquisition are strong, questions still remain on optimising the user selection, the ideal distribution platform and optimal monitoring schedule. Ongoing health service research aims to address these knowledge gaps. For more information, consult the AVAC website http://www.avac.org/prevention-option/prep.

Please report adverse events occurring on PrEP to the National Adverse Drug Event Monitoring Centre, which is housed in the Division of Pharmacology at the University of Cape Town. The reporting guideline is available at: http://www.mccza.com/genericDocuments/2.11_ADR_reporting_Jun11_v2.doc.
